# Oligo Hyaluronan‐Coated Silica/Hydroxyapatite Degradable Nanoparticles for Targeted Cancer Treatment

**DOI:** 10.1002/advs.201900716

**Published:** 2019-04-30

**Authors:** Yao Kang, Wen Sun, Shuyi Li, Mingle Li, Jiangli Fan, Jianjun Du, Xing‐Jie Liang, Xiaojun Peng

**Affiliations:** ^1^ State Key Laboratory of Fine Chemicals Dalian University of Technology Dalian 116024 China; ^2^ Research Institute of Dalian University of Technology in Shenzhen Gaoxin South fourth Road, Nanshan District Shenzhen 518057 China; ^3^ CAS Key Laboratory for Biomedical Effects of Nanomaterials and Nanosafety CAS Center for Excellence in Nanoscience National Center for Nanoscience and Technology of China Beijing 100190 China

**Keywords:** chemotherapy, degradable nanoparticles, MSNs/HAP, oligo hyaluronan, tumor inhibition

## Abstract

Targeted drug delivery systems (TDDSs) provide a promising approach to overcome the side effect of traditional chemotherapy by specific tumor targeting and drug release. Hyaluronan (HA), as a selective CD44 targeting group, has been widely used in TDDSs for chemotherapy. However, different molecular weight HAs would demonstrate different binding ability to CD44, which may result in different therapeutic effects. Herein, a silica/hydroxyapatite (MSNs/HAP) hybrid carrier loaded with anticancer drug doxorubicin (DOX) (DOX@MSNs/HAP) is fabricated. HA and oligo HA (oHA) are coated onto the nanoparticles (HA‐DOX@MSNs/HAP, oHA‐DOX@MSNs/HAP), respectively, to investigate their performance in tumor targeting ability. oHA‐DOX@MSNs/HAP shows much higher efficiency cellular uptake and drug release in tumor regions due to more effective CD44 targeting of oHA. Thus, the anticancer effect of oHA‐DOX@MSNs/HAP is significantly enhanced compared to HA‐DOX@MSNs/HAP, as demonstrated in a tumor‐bearing mouse model. This study may enable the rational design of nanodrug systems for future tumor‐targeted chemotherapy.

## 1 Introduction

In cancer therapies, the cellular uptake efficiency of therapeutic drugs is the key factor determining the result of cancer treatment.^1^, ^2^, ^3^, ^4^, ^5^, ^6^, ^7^, ^8^ Free anticancer drugs decentralize to the entire body with low accumulation in tumors, which results in low therapeutic efficiency and serious toxic side effects.^9^, ^10^, ^11^ In contrast, targeting drug delivery systems (TDDSs) that have been widely used in cancer treatment provide a promising approach to improve the specificity of therapy, and minimize the undesired side effects to healthy tissues.^1^, ^4^, ^8^, ^12^, ^13^, ^14^, ^15^, ^16^, ^17^, ^18^

An important factor for TDDSs is the ability to target cancer cells.^19^, ^20^, ^21^ Generally, many overexpressed receptors in cancer cells provide a well‐defined strategy for selectively targeting cancer cells.^22^, ^23^, ^24^ CD44 is a transmembrane cell surface glycoprotein that is endogenously expressed at low levels on various cell types in normal tissues, but highly expressed on various types of cancer cells, including breast, pancreas, colon, prostate, and stomach cancer, determining their tumorigenic and metastatic capacities.^25^, ^26^, ^27^ As an essential component of the extracellular matrix, hyaluronan (HA) is a large, nonsulphated glycosaminoglycan composed of a repeating disaccharide unit of *N*‐acetyl‐D‐glucosamine and D‐glucuronic acid.^28^, ^29^, ^30^ HA possesses affinity for CD44 receptors and thus can be utilized as a targeting ligand for tumors.^31^ Generally, HA with molecular weight of >20 KDa can be cleaved into oligosaccharides (oHA < 10 KDa) under HAase as a “molecular saboteur” in biosystems.^32^, ^33^, ^34^, ^35^ Accordingly, some TDDSs containing HA as the tumor‐targeting groups have been developed to inhibit tumor growth, in most of which HA was adopted.^36^, ^37^, ^38^, ^39^, ^40^, ^41^, ^42^ For instance, Yin and co‐workers reported a HA−hydrophobic‐active (HA: 1.5 MDa) liposomes prodrug that can be self‐assembled into a smart nanoplatform for targeting tumor cells and combined treatment.^37^ Ossipov and co‐workers reported a HMW HA‐bisphosphonate conjugate (HA: 1.3 MDa) having free hydrazide functionality that can be used as an antiosteoclastic and antineoplastic drug in the injectable hydrogel formulation.^41^ Nairi et al. prepared mesoporous silica nanoparticles (MSNs) functionalized with HA of different chain length for cancer targeted drug delivery in vitro.^42^ However, HA‐coated TTDSs always display limited tumor targeting ability and finally resulted in inefficient cancer treatment.^43^ In fact, native HA and oHA provoke distinct biological effects upon binding to CD44.^35^, ^44^ HA binding to CD44 selectively induces CD44 clustering, suggesting that the delivered drug would be adhered to the cancer cell membrane and thus might not easily internalize into the cells.^45^, ^46^ In addition, CD44 clustering can also increase the exocytosis of inner hyaluronan (HA, HA fragments, and oHA).^45^ On the contrary, the clustering of CD44 could be inhibited by oHA and the reversible binding would make TDDSs cell permeable for efficient cellular uptake.^47^, ^48^ Therefore, it is of great importance to study the effect of oHA on tumor targeting and anticancer effect in vivo.

On the other hand, mesoporous silica nanoparticles (MSNs) are among the most widely used materials in the construction of TDDSs due to their extremely large surface area for efficient drug loading.^49^, ^50^, ^51^, ^52^, ^53^ However, the poor degradability of MSNs may result in long‐term toxicity, which limits their future clinic transitions.^54^, ^55^, ^56^ Complementary to silica, as the most important constituent of bones, hydroxyapatite (HAP) features excellent biocompatibility and nontoxicity to biosystems.^57^, ^58^, ^59^, ^60^ More importantly, it is sensitive to pH and easy to be degraded under weak acidic cancer cell pH (6.5–6.8).^61^, ^62^ Therefore, the hybrid materials via the incorporation of HAP into MSNs may not only allow the pH‐induced degradability but also improve the drug loading amount, drug release efficiency, and therapeutic outcomes.

**Scheme 1:**
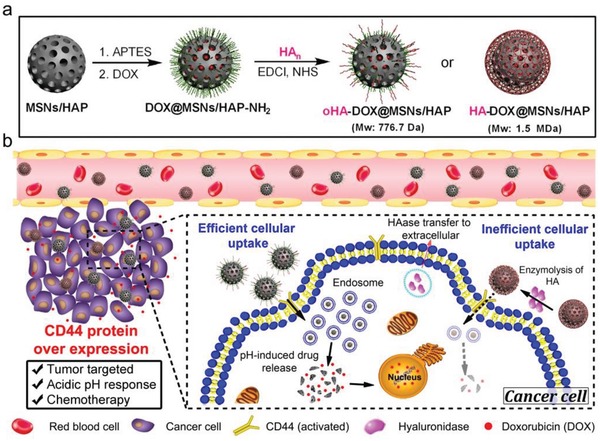
a) Preparation scheme of oHA‐DOX@MSNs/HAP and HA‐DOX@MSNs/HAP nanoparticles. b) Working principles of oHA‐DOX@MSNs/HAP and HA‐DOX@MSNs/HAP as nanodrugs for targeted chemotherapy in vivo.

Herein, we fabricated a MSNs/HAP hybrid drug carrier and loaded anticancer drug doxorubicin (DOX) in the mesoporous of silica framework (**Scheme** 1 and Scheme S1, Supporting Information). For the first time, HA and oHA were coated on the DOX@MSNs/HAP nanoparticles, respectively, to investigate their performance in tumor targeting ability. Consequently, both oHA‐DOX@MSNs/HAP and HA‐DOX@MSNs/HAP nanoparticles showed ideal bio‐safety to normal cells (COS‐7 cells). However, due to more efficient targeting ability of oHA than HA, oHA‐DOX@MSNs/HAP nanoparticles displayed much better cellular uptake efficacy and released drug in cancer cells compared to HA‐DOX@MSNs/HAP. Moreover, oHA‐DOX@MSNs/HAP nanoparticles demonstrate specific tumor targeting, and excellent tumor inhibiting effects with ignorable toxic side effects in living mice. Overall, the comparison in this study reveals the impact of molecular weight of HA in tumor targeting effect, in which the oHA coated nanoparticles show better chemotherapy properties due to its reversible binding ability to overexpressed CD44 on cancer cell membrane, thus demonstrating a constructive suggestion in nanodrug design and offering an efficient strategy for tumor‐targeted chemotherapy.

## 2 Results and Discussion

**Figure 1:**
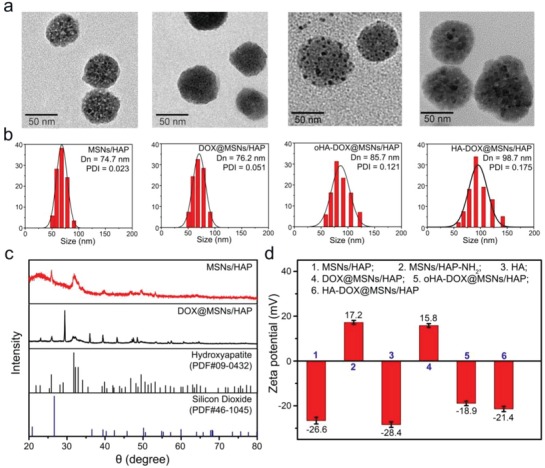
a) TEM images and b) DLS analysis of MSNs/HAP, DOX@MSNs/HAP, oHA‐DOX@MSNs/HAP, and HA‐DOX@MSNs/HAP; c) XRD spectra of MSNs/HAP, DOX@MSNs/HAP, and standard cards of hydroxyapatite (PDF#09‐0432) and silicon dioxide (PDF#46‐1045); d) zeta potential changes after functionalization.

The synthesis of HA‐coated hybrid nanodrug involved a multistep nanoscale particle formation and surface modifications (Scheme 1a). The mesoporous MSNs/HAP hybrid skeleton was initially fabricated via a hydrothermal approach. Amino groups were further functionalized onto the surface by using (3‐aminopropyl)triethoxysilane (APTES) as a coupling reagent, followed by loading anticancer drug DOX into the mesoporous of MSNs/HAP nanoparticles (37.21% of drug loading efficiency). oHA (MW = 776.7 Da) and HA (MW = 1.5 MDa) were grafted covalently to DOX@MSNs/HAP nanoparticles, respectively (2.17% and 4.91% for grafting capacity of HA). Each step of the as‐obtained nanoparticles were well characterized by transmission electron microscopy (TEM), scanning electron microscope (SEM), dynamic light scattering (DLS), Fourier transform infrared spectroscopy (FT‐IR), and X‐ray diffraction spectra (XRD). MSNs/HAP was spherical with a uniform size distribution and clear mesoporous in the structure (**Figure** 1 and Figure S1, Supporting Information). While, after drug loading and surface modification with HA, the mesoporous disappeared obviously. DLS measurements revealed that both MSNs/HAP and DOX@MSNs/HAP were monodisperse in a narrow size distribution with a mean diameter of 75 nm (Figure 1b). As expected, the diameters increased to 85 nm (oHA‐DOX@MSNs/HAP) and 100 nm (HA‐DOX@MSNs/HAP) after modification with oHA or HA, respectively, which agreed well with the TEM observations. Further, the nanoparticles expressed promising dispersion stability with low PDI values during two weeks (Figure S2, Supporting Information). The XRD analysis of MSNs/HAP and DOX@MSNs/HAP were also conducted (Figure 1c). These peaks in the region between 20° and 35° of MSNs/HAP were assigned to the hexagonal hydroxyapatite (PDF#09‐0432), indicating that the hydroxyapatite was successfully embedded into the skeleton of MSNs. A typical MSN peak at 27° was also clearly detected in the spectrum of MSNs/HAP. Conversely, these abovementioned peaks significantly decreased after drug‐loading. Zeta potential changes guided each step of the nanoparticles synthesis, in which positive potentials (17.2 and 15.8 mV) were measured after surface amino functionlization, and negative potentials were detected after chemically linking with oHA (−18.9 mV) and HA (−21.9 mV) (Figure 1d). As FT‐IR spectra shown in Figure S3 in the Supporting Information, distinct changes occurred during the preparation process. Two new bands at 2924 and 2973 cm^−1^ assigned to the N–H stretching vibration emerged in MSNs/HAP‐NH_2_, indicating that the amino groups were successfully grafted to the MSNs/HAP nanoparticles surface. The reduced intensity at 1076 and 897 nm^−1^ attributed to Si–O–Si vibrations and O–Si–O bending vibrations indicated the success DOX loading into the nanostructure. Furthermore, the energy dispersive X‐ray spectroscopy (EDX) also demonstrated the existence of Si, O, Ca, and P within the nanostructure (Table S1, Supporting Information). Besides, as determined from N_2_ adsorption–desorption isotherm measurements, MSNs/HAP exhibited a high surface area of ≈650 m^2^ g^−1^ with pore sizes of 2.75 and 14.26 nm, originated from MSNs and HA, respectively (Figure S4, Supporting Information). However, the surface area decreased sharply to 27.12 m^2^ g^−1^ and nearly no pores could be detected in DOX@MSNs/HAP, indicative of the drug loading inside of the nanostructure mesoporous. Additionally, DOX fluorescence was significantly quenched in DOX@MSNs/HAP, which further confirmed the successful drug loading into the nanoparticles (Figure S5, Supporting Information).

**Figure 2:**
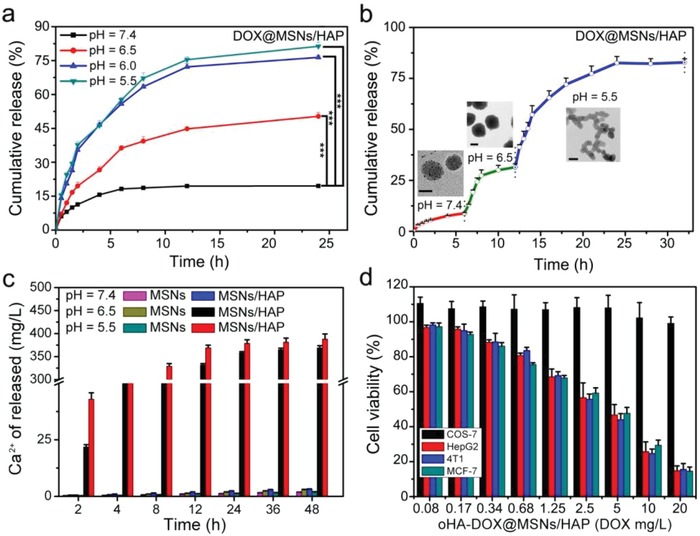
a) Release profiles of DOX from DOX@MSNs/HAP in PBS buffer with different pH values (*p* < 0.001, Tukey's multiple comparison test). b) Step release DOX from DOX@MSNs/HAP in PBS buffer at different pH values. Inset TEM images were taken at 4, 10, and 24 h incubation, respectively. c) Ca^2+^ release from MSNs and MSNs/HAP at pH = 7.4, 6.5, and 5.0 after different incubation time (*n* = 5). d) Viability of normal cells (COS‐7) and cancer cells (HepG2, MCF‐7 and 4T1) treated with oHA‐DOX@MSNs/HAP at various concentrations (*n* = 3).

The controlled release of loaded drugs at tumor regions is a key performance indicator of nanoparticles in cancer treatment. We assessed DOX release from DOX@MSNs/HAP, oHA‐DOX@MSNs/HAP, and HA‐DOX@MSNs/HAP nanoparticles at different pH conditions (7.4, 6.5, 6.0, 5.5) (**Figure** 2a and Figure S6, Supporting Information). Less than 15% of DOX was released even after incubation for 24 h in pH = 7.4 neutral environment, indicating the high stability of DOX@MSNs/HAP. The low drug leakage can significantly reduce the toxic side effects to healthy tissues. Conversely, in acidic environment, DOX release from DOX@MSNs/HAP dramatically increased to 50%, 76%, and 81% at pH 6.5, 6, and 5.5, respectively. Similar results of DOX release were obtained from oHA‐DOX@MSNs/HAP and HA‐DOX@MSNs/HAP nanoparticles at different pH, suggesting that the surface‐drafted HA and oHA cannot block DOX release. We further investigated the relationship between drug release and nanoparticle morphology changes in different pH conditions via three steps: step 1, pH = 7.4 for 6 h, step 2: pH = 6.5 for 6 h, and step 3: pH = 5.5 for 24 h (Figure 2b). The DOX release profile and TEM images of the nanoparticles (Figure 2b) were recorded simultaneously in each step. In neutral environment (pH 7.4), the nanoparticles were intact with limited drug release, while in acidic conditions (pH 6.5 and 5.5), they degraded gradually into smaller nanostructures to release DOX. Thus, the MSNs/HAP nanoparticles have the potential to release DOX at tumor sites due to their weak acidic environment (pH 6.4–6.8). The acid‐induced DOX release was attributed to the hybrid HAP in silica structure, resulting in the degradation of the nanoparticles. In addition, we also measured Ca^2+^ release from the hybrid nanoparticles by complexometric titration with EDTA and eriochrome black T (Figure 2c). A large content of Ca^2+^ was detected in the acidic solution (both 5.5 and 6.5) which further demonstrated their degradation.

The cytotoxicity of nanoparticles to both cancer and normal cells were accessed (Figure 2d and Figure S7, Supporting Information). Cells were incubated with oHA‐DOX@MSNs/HAP and HA‐DOX@MSNs/HAP with different concentrations, and cell viabilities were evaluated after 24 h incubation. Both oHA‐DOX@MSNs/HAP and HA‐DOX@MSNs/HAP were almost nontoxic to normal cells due to inefficient cellular uptake by normal cells and nontoxicity of the nanocarriers (Figure S8, Supporting Information). Conversely, upon incubation with HA or oHA coated nanoparticles, the viabilities of cancer cells sharply decreased due to the HA or oHA‐mediated cellular uptake and intracellular pH‐induced drug release. Furthermore, compared with HA‐DOX@MSNs/HAP, oHA‐DOX@MSNs/HAP exhibited more toxicity to cancer cells; the IC_50_ of oHA‐DOX@MSNs/HAP to HepG2, MCF‐7, and 4T1 cancer cells were 4.04, 3.84, 4.44 mg DOX per L respectively, which were much lower than that of HA‐DOX@MSNs/HAP (7.46, 7.06, 7.55 mg DOX per L) (Table S2, Supporting Information). The cell death of 4T1 caused by MSNs/HAP, DOX@MSNs/HAP, HA‐DOX@MSNs/HAP, and oHA‐DOX@MSNs/HAP was also evaluated after long‐time incubation (60 h) to further demonstrate the efficient anticancer effect of oHA‐DOX@MSNs/HAP (Figure S9, Supporting Information). As expected, oHA‐DOX@MSNs/HAP still exhibited the most efficient inhibiting effect. These results demonstrated that HA or oHA coating allowed the hybrid nanoparticles specifically target CD44 over‐expressed cancer cells and resulted in cytotoxicity, and oHA further enhanced the toxicity to cancer cells.

**Figure 3:**
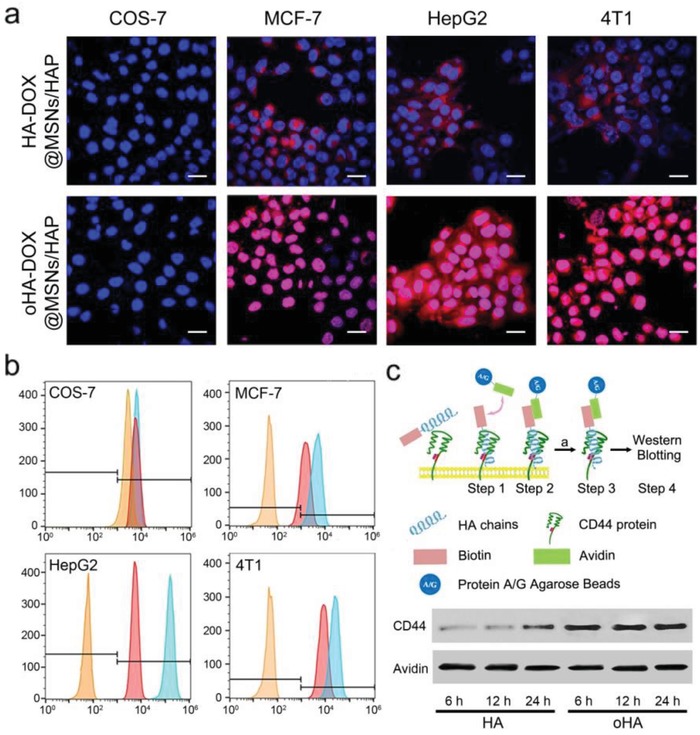
a) CLSM images of cancer cells after incubation with HA‐DOX@MSNs/HAP or oHA‐DOX@MSNs/HAP for 4 h. Blue: Hoechst 33342 (λ_ex_ = 405 nm, λ_em_ = 430–470 nm); Red: DOX (λ_ex_ = 488 nm, λ_em_ = 590–620 nm); Purple: merged with blue and red. Scale bar: 20 µm. b) CD44 expression levels of cancer and normal cells after different treatments by using CD44‐PE monoclonal antibody. Blue: control; Red: incubation with HA‐DOX@MSNs/HAP for 12 h; Yellow: incubation with oHA‐DOX@MSNs/HAP for 12 h. c) Schematic illustration of “pull‐down” to study the interactions between oHA/HA with CD44 by using specific biotin and avidin interaction. Western blotting results indicate that oHA shows stronger interaction with CD44 than HA.

We studied the cellular uptake of the hybrid nanoparticles based on confocal laser scanning microscopy (CLSM) via monitoring the DOX fluorescence (**Figure** 3a and Figure S10, Supporting Information). These nanoparticles were incubated with three cancer cell lines including human breast cancer cells (MCF‐7, CD44 normal expressed), mouse epithelial breast cancer cells (4T1, CD44+, over‐expressed), human epithelial liver cancer cells cancer cells (HepG2, CD44+, over‐expressed), and normal cercopithecus aethiops kidney cells (COS‐7, CD44 in resting state) for 4 h. Subsequently, cells were washed thoroughly and cell nuclei were stained with Hoechst 33342. As shown in Figure 3a and Figure S10 in the Supporting Information, nearly no red fluorescence from DOX was observed in the nucleus of COS‐7, indicating that both oHA‐DOX@MSNs/HAP and HA‐DOX@MSNs/HAP were not taken up by normal cells. In contrast, these nanoparticles were capable of internalizing into cancer cells after incubation, since clear red fluorescence in nucleus was detected from cancer cells. It was worth noting that oHA‐coated nanoparticles (oHA‐DOX@MSNs/HAP) exhibited much more efficient cellular uptake compared to HA‐DOX@MSNs/HAP, as higher fluorescence intensity was observed after incubating cancer cells with oHA‐DOX@MSNs/HAP. In addition, no DOX fluorescence was detected from the control group after nucleus being labelled with Hoechst 33342, which indicated that Hoechst 33342 did produce unexpected interference; DOX fluorescence indeed came from the DOX‐loaded nanoparticles (Figure S11, Supporting Information). To further study the difference in cellular uptake between HA‐DOX@MSNs/HAP and oHA‐DOX@MSNs/HAP, CD44 monoclonal antibody was used to preincubate with cancer cells before incubating with nanoparticles. All cells after being pretreated with CD44 monoclonal antibody demonstrated inefficient cellular uptake of nanoparticles, which indicated that the distinct uptake of nanoparticles are mediated by CD44 on cell membrane (Figure S12, Supporting Information).

The cellular uptake was also studied via the detection of intercellular HA by using alcan blue after incubation with the hybrid nanoparticles. 4T1 cells were first incubated with oHA‐DOX@MSNs/HAP or HA‐DOX@MSNs/HAP for 24 h. Then the cell suspensions were washed by PBS buffer to remove the free nanoparticles from cell culture. After centrifugation, cell lysis was carried out followed by staining with alcian blue. In fact, alcian blue, a copper‐titanium blue conjugate dye, is a commercial colorant and used for the detection of mucopolysaccharides via interacting with acidic groups of mucopolysaccharides to induce the absorption change. As shown in Figure S13 in the Supporting Information, cells after incubation with oHA‐DOX@MSNs/HAP displayed a higher level of absorption intensity, indicating that oHA resulted in much more efficient cellular uptake. Therefore, oHA‐DOX@MSNs/HAP nanoparticles with oHA coating were expected to be more efficient for anticancer treatment.

To further study the tumor targeting ability of HA, a series of flow cytometry (FCM) (Figure 3b, Figure S14 and Table S3, Supporting Information) and “pull‐down” experiments (Figure 3c) were carried out. After incubating cells with oHA@MSNs/HAP or HA@MSNs/HAP, the CD44‐PE (phycoerythrin) monoclonal antibody was applied to analysis the content of free CD44 protein in different cell lines including COS‐7, MCF‐7, HepG2, and 4T1. As shown in Figure S14 in the Supporting Information, the interaction between oHA and CD44 is stronger than that of HA because of the distinct fluorescence intensity decrease observed from oHA treated cancer cells. We then studied the combination between HA or oHA‐coated MSNs/HAP and CD44. The abovementioned cells were incubated with oHA@MSNs/HAP and HA@MSNs/HAP, respectively. Similarly, both oHA@MSNs/HAP and HA@MSNs/HAP demonstrated nearly no influence on normal cell COS‐7 (Figure 3b). Compared with normal cells, fluorescence changes can be observed from cancer cells after incubating with these nanoparticles (Table S3, Supporting Information). HA@MSNs/HAP moderately decreased the fluorescence due to a weak combination of HA to CD44. In contrast, the PE fluorescence from cancer cells significantly decreased in the presence of oHA@MSNs/HAP, suggesting that a large amount of CD44 interacted with the surface grafted oHA. The results demonstrated that oHA@MSNs/HAP had a stronger interaction toward CD44.

The protein pull‐down assay based on biotin/avidin interaction was applied to investigate the combination between oHA/HA and CD44 (Figure 3c and Figure S15, Supporting Information). In this case, biotin labeled oHA (or HA) was synthesized, followed by incubating with cells lysis for 6, 12, and 24 h to get biotin‐HA‐CD44 complexes. Then, an agar gel (labeled with avidin) was added into the above mixture to form A/G‐avidin‐biotin‐HA‐CD44, allowing the measurement of CD44 content via western blotting analysis. Obviously, this approach directly studied the combination between HA and CD44 with avoidance of many other factors including diameter of nanoparticles, drug release, and destructive process of cell lysis. The CD44 lanes of oHA displayed much higher intensity than that of HA, indicating that oHA showed stronger interaction with CD44, which could result in more efficient cellular uptake by CD44‐overexpressed cancer cells (Figure 3c and Figure S15, Supporting Information).

**Figure 4:**
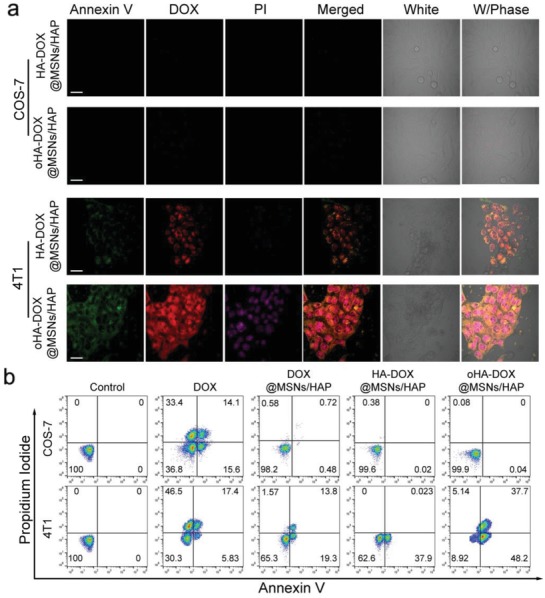
a) CLSM images of cellular apoptosis of COS‐7 and 4T1 cells after incubation with oHA‐DOX@MSNs/HAP or HA‐DOX@MSNs/HAP for 12 h via the AV‐PI (Annexin V‐FITC and Propidium Iodide) staining. Scale bar: 20 µm. b) Flow cytometric examination of apoptosis of COS‐7 and 4T1 cells after different treatments for 24 h.

The toxicities of the hybrid nanoparticles to cancer cells were also studied via the AV‐PI (Annexin V‐FITC and Propidium Iodide) staining, which are applied for imaging early apoptosis and dead cells, respectively (**Figure** 4, Figures S16 and S17, Supporting Information). Therefore, matching AV‐PI can distinguish different stages of cell apoptosis. Nearly no green (AV) and purple fluorescence (PI) were detected in the control groups (COS‐7, HepG2, and 4T1 cells), suggesting that AV‐PI kits did not bring interference; cell apoptosis was indeed caused by the nanoparticles (Figure S16, Supporting Information). In contrast, obvious green fluorescence was detected in 4T1 cells after treatment with HA‐DOX@MSNs/HAP for 12 h due to moderate cellular uptake and low pH‐induced drug release, which demonstrated that HA‐DOX@MSNs/HAP can induce early cell apoptosis (Figure 4a). It is notable that oHA‐DOX@MSNs/HAP brought the strongest green and red fluorescence, which validated that oHA allowed the more efficient cellular uptake and enabled the reinforcement on inducing apoptosis of cancer cell. Furthermore, FCM study performed on COS‐7, 4T1, and HepG2 cells after different treatments were conducted (Figure 4b and Figure S17, Supporting Information). As shown in Figure S16 in the Supporting Information, cells that were treated with free DOX showed a high percent in the necrotic stage, suggesting its toxic side effect to both cancer and normal cells. On the contrary, DOX@MSNs/HAP and HA‐coated DOX@MSNs/HAP demonstrated nontoxic to normal cells due to their excellent biocompatibility and high stability under neutral conditions. oHA‐DOX@MSNs/HAP showed the most efficient anticancer effect; more than 85% cancer cells were in the apoptosis stage and no cell necrosis occurred after treatment. These results indicated that oHA‐DOX@MSNs/HAP realized efficient internalization into cancer cells and intracellular drug delivery, enhanced the anticancer efficacy, and simultaneously reduced the toxic side effects to normal cells.

**Figure 5:**
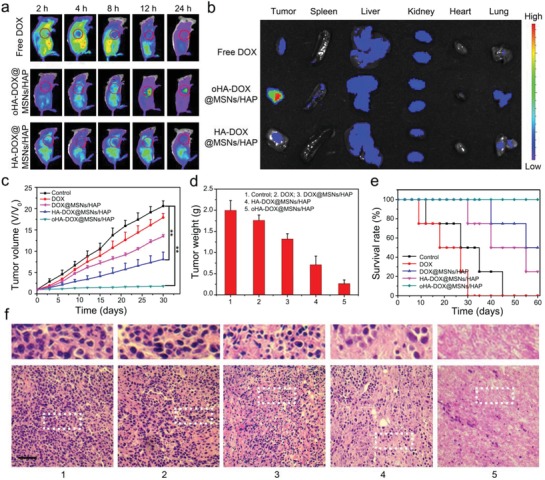
a) In vivo fluorescence imaging of 4T1 tumor‐bearing mice after intravenous injection of DOX and oHA‐DOX@MSNs/HAP. Images were taken at 2, 4, 8, 12, and 24 h after injection. b) Ex vivo fluorescence imaging of important organs and the tumor tissues (1. heart; 2. liver; 3. lung; 4. stomach; 5. spleen; 6. kidney; 7. tumor). Organs and tumor tissues were collected 24 h postinjection of oHA‐DOX@MSNs/HAP or DOX. (*p* < 0.005, Tukey's multiple comparison test). c) The relative tumor volume of tumor‐bearing mice during different treatments (*n* = 4). d) Average weights of tumors after treatment (*n* = 4). e) Survival rates of mice after different treatments. f) H&E staining images of tumor tissues of each group after treatment (1. Control; 2. DOX; 3. DOX@MSNs/HAP; 4. HA‐DOX@MSNs/HAP; 5. oHA‐DOX@MSNs/HAP). Scale bar: 100 µm.

The efficient anticancer performance of oHA‐DOX@MSNs/HAP in vitro motivated us to investigate the tumor inhibition using the nanoparticles in vivo. 4T1 tumor‐bearing mice were treated with free DOX, oHA‐DOX@MSNs/HAP, and HA‐DOX@MSNs/HAP via intravenous injection through the tail vein. After injection for 2, 4, 8, 12, and 24 h, the biodistribution of free DOX and the nanoparticles were monitored by optical imaging performed on an in vivo imaging system (**Figure** 5a,b). After injection of free DOX, the drug distributed in the whole body at the beginning and cleared out rapidly through blood stream. In contrast, obvious fluorescence signal was detected in the tumor site in the other two groups after 12 h, in which the oHA‐DOX@MSNs/HAP group expressed the most efficient tumor accumulation (Figure 5b). Prolonged circulation can help to achieve sufficient accumulation of nanoparticles in tumor regions. Thus, the circulation of oHA‐DOX@MSNs/HAP was further investigated. The Si contents in blood were quantified by inductively coupled plasma mass spectrometry (ICP‐MS). As shown in Figure S18 in the Supporting Information, even after injection of the nanoparticles for 48 h, the Si concentration in blood was still above 3.40% dose per mL of blood. The results demonstrated oHA‐coated nanoparticles exhibit prolonged circulation in vivo, thus resulting in efficient and long‐term tumor‐targeted accumulation. The tumor and other organs including heart, liver, lung, stomach, spleen, and kidney were further isolated for fluorescence imaging (Figure S19, Supporting Information). Strong fluorescence of oHA‐DOX@MSNs/HAP was observed from the tumor site, while only low fluorescence intensity was found from the tumor in free DOX and HA‐DOX@MSNs/HAP groups. Furthermore, the distribution of oHA‐DOX@MSNs/HAP nanoparticles in living mice was also conducted via ICP‐MS analysis of Si. The concentration of Si in tumors was much higher than that of other organs (Figure S20, Supporting Information). Therefore, oHA‐DOX@MSNs/HAP nanoparticles showed excellent tumor accumulation with high specificity, which greatly enhanced the bioavailability of DOX to tumors.

We evaluated the in vivo antitumor efficacy of oHA‐DOX@MSNs/HAP nanoparticles on a 4T1 tumor‐bearing mouse model. The mice were divided into five groups and treated differently through intravenous injection of saline, free DOX (5 mg kg^−1^), DOX@MSNs/HAP (5 mg DOX per kg), HA‐DOX@MSNs/HAP (5 mg DOX per kg), and oHA‐DOX@MSNs/HAP (5 mg DOX per kg). The tumor volume of each group was recorded every three days during a 30 day period. The tumor growth inhibition efficiencies were 17.96 ± 0.047%, 38.09 ± 0.014%, 62.80 ± 0.076%, and 91.79 ± 0.010% for free DOX, DOX@MSNs/HAP, HA‐DOX@MSNs/HAP, and oHA‐DOX@MSNs/HAP, respectively (Figure 5c). Although DOX@MSNs/HAP inhibited the tumor growth to some extent, oHA‐DOX@MSNs/HAP brought about the strongest tumor growth suppression and nearly no weight loss after treatment (Figure S21, Supporting Information), indicating the importance of oHA for tumor targeting. The tumor weight and the representative tumor images in each group were in accordance with the result of relative tumor volumes (Figure 5d and Figure S22, Supporting Information). In addition, the histological analysis of the tumor tissues was carried out to evaluate the antitumor efficacy of different treatments (Figure 5f). As shown from hematoxylin and eosin (H&E) staining of tumor tissues, no obvious destruction was detected in the PBS group, whereas the tissues in the free DOX, DOX@MSNs/HAP, HA‐DOX@MSNs/HAP, or oHA‐DOX@MSNs/HAP groups experienced different levels of changes, with a certain amount of cells in the apoptotic state. Clearly, the highest level of damage was observed in the oHA‐DOX@MSNs/HAP group, which evidenced the promising tumor ablation activity of the oHA coated nanoparticles.

During the treatment period, the mice treated with PBS, DOX@MSNs/HAP, HA‐DOX@MSNs/HAP, or oHA‐DOX@MSNs/HAP did not show any abnormal behavior. Histological analysis of the major organs (heart, liver, lung, stomach, spleen, and kidney) was performed (Figure S23, Supporting Information). No noticeable pathological changes or damages were found in the main organ slices from the H&E staining images of DOX@MSNs/HAP, oHA‐MSNs/HAP, and oHA‐DOX@MSNs/HAP groups, confirming that the hybrid material eliminated substantial systemic toxicity. While, myocardial fiber breakage was observed in the free DOX group due to its high toxicity to normal tissues. Moreover, all mice treated with DOX died during the treatment period. In contrast, all mice treated with oHA‐DOX@MSNs/HAP survived after 2 months (Figure 5e), which was also superior to the other three groups with low survival rates (0% for PBS, 25% for DOX@MSNs/HAP, 50% for HA‐DOX@MSNs/HAP). Additionally, these nanoparticles were biocompatible to blood, as the blood hemolysis did not occur when blood cells were incubated with the DOX@MSNs/HAP, HA‐DOX@MSNs/HAP, and oHA‐DOX@MSNs/HAP (Figure S24, Supporting Information). Serum CK level (creatine kinase) is an important biochemical index; abnormal high serum CK means metabolic disturbance and tissue damage. As shown in the Figure S25 in the Supporting Information, nearly no change was observed from mice treated with oHA‐DOX@MSNs/HAP, HA‐DOX@MSNs/HAP, or DOX@MSNs/HAP, suggesting that the injection of the hybrid nanoparticles did not cause side or adverse effects to serum. However, serum CK of the free DOX group was significantly increased due to toxicity of the free drug. Taken together, these results demonstrated that oHA‐DOX@MSNs/HAP nanoparticles improved DOX availability to tumors, enhanced the antitumor efficacy, and reduced its systemic toxicity via HA involved passive tumor targeting.

## 3 Conclusion

In summary, we successfully developed MSNs/HAP hybrid drug carriers and loaded DOX in the mesoporous of silica framework. The hybrid structure allowed the nanoparticles have both advantages of MSNs and HAP, resulting in improved drug loading amount and pH‐responsiveness for drug release. oHA and HA were coated on the DOX@MSNs/HAP nanoparticles, respectively, to investigate their performance for tumor targeting. Compared to HA, oHA could inhibit the clustering of CD44 and then make the hybrid materials cell permeable for more efficient uptake by cancer cells. Thus, it is found that oHA‐DOX@MSNs/HAP nanoparticles displayed much better cellular uptake efficacy than HA‐DOX@MSNs/HAP. Furthermore, oHA‐DOX@MSNs/HAP nanoparticles demonstrate efficient tumor accumulation and tumor inhibiting effects. The biosafety tests showed that the alternative of the oHA coated MSNs/HAP drug carrier can eliminate substantial systemic toxicity and thus attract in vivo application prospects. The study reveals the impact of molecular weight of HA on tumor targeting effect in nanocarriers, providing consultations for designing other drug delivery systems for tumor‐targeted chemotherapy in the future.

## Conflict of Interest

The authors declare no conflict of interest.

## Supporting information

Supplementary
