# Supplementary material for: Oligo Hyaluronan‐Coated Silica/Hydroxyapatite Degradable Nanoparticles for Targeted Cancer Treatment
Source: Adv Sci (Weinh). 2019 Apr 30;6(13):1900716. doi: 10.1002/advs.201900716 (PMC6662421; doi:10.1002/advs.201900716)
Supplement: Supplementary file 1 — Supplementary [file ADVS-6-1900716-s001.pdf]

Copyright WILEY-VCH Verlag GmbH & Co. KGaA, 69469 Weinheim, Germany, 2019.

ADVANCED SCIENCE
Open Access

Supporting Information

for *Adv. Sci.,* DOI: 10.1002/advs.201900716

Oligo Hyaluronan-Coated Silica/Hydroxyapatite Degradable Nanoparticles for Targeted Cancer Treatment

Yao Kang, Wen Sun, Shuyi Li, Mingle Li, Jiangli Fan, Jianjun Du, Xing-Jie Liang,* and Xiaojun Peng*

Copyright WILEY-VCH Verlag GmbH & Co. KGaA, 69469 Weinheim, Germany, 2019.

Supporting Information

Oligo Hyaluronan-coated Silica/Hydroxyapatite degradable nanoparticles for Targeted Cancer Treatment

Yao Kang, Wen Sun, Shuyi Li, Mingle Li, Jiangli Fan, Jianjun Du, Xing-Jie Liang*, Xiaojun Peng*

Y. Kang, Dr. W. Sun, M. Li, Prof. J. Fan, Prof. J. Du, Prof. X. Peng
State Key Laboratory of Fine Chemicals, Dalian University of Technology, Dalian, 116024, China.
E-mail: fanjl@dlut.edu.cn

Dr. S. Li, Prof. X. Liang
CAS Key Laboratory for Biomedical Effects of Nanomaterials and Nanosafety, CAS Center for Excellence in Nanoscience, National Center for Nanoscience and Technology of China, Beijing 100190, China
E-mail: liangxj@nanoctr.cn

Dr. W. Sun, Prof. J. Fan, Prof. J. Du, Prof. X. Peng
Research Institute of Dalian University of Technology in Shenzhen, Gaoxin South fourth Road, Nanshan District, Shenzhen 518057, China

Materials. Hyaluronic acid (oHA, MW 776 Da; HA, 1.5 MDa), Cetyltrimethylammonium bromide (CTAB), tetraethyoxysilane (TEOS), 3-(4,5)-dimethylthiahiazo(-2-yl)-3,5-diphenyltetrazolium bromide (MTT), 2-(7-aza-1H-benzotriazole-1-yl)-1,1,3,3-tetrame hyluroniumhexafluoro-phosph ate (HATU) malononitrile (99%), aminopropyl triethoxysilane (APTES), 6-nitrobenzo[de]isochromene-1,3-dione (98%), o-phenylenediamine (99%) and N,N-diisopropylethyl amine (DIPEA, 99.5%) were purchased from Energy Chemical

Co., Ltd. (Shanghai, China). Calcium nitrate [$Ca(NO_3)_2$], diammonium hydrogen phosphate [$(NH_4)_2HPO_4$)], Alcian Blue 8GX were purchased from Aladdin® Co., Ltd. (Beijing, China). Doxorubicin hydrochloride (DOX) was purchased from Nanjing Dilger Medical Technology Co., Ltd (Nanjing, China). DMEM medium and penicillin-streptomycin solution were purchased from HyClone (Logan City, USA). Fetal bovine serum (FBS) was purchased from PAN-biotech (Aidenbach, Germany). Hoechst 33342 and Annexin V-FITC/PI staining kits were purchased from the Nanjing KeyGEN Biotech Co., Ltd. (Nanjing, China). All other chemicals were of analytical grade and were used without further purification.

Cell culture. 4T1 (cancer cell, epithelial, breast adenocarcinoma cell, provided by Shanghai Institute of Biochemistry and Cell Biology), MCF-7 (cancer cell, epithelial, breast adenocarcinoma cell, provided by Shanghai Institute of Biochemistry and Cell Biology), HepG2 (cancer cell, epithelial, hepatocellular carcinoma cell, provided by KeyGEN Biotech Co. Ltd), and COS-7 (normal cell, African green monkey kidney cell, provided by KeyGEN Biotech Co. Ltd) cells were used in this work. The above four cell lines were incubated with DMEM or RPMI 1640 medium (HyClone), containing 10% fetal bovine serum (PAN), 0.03% L-glutamine, penicillin and streptomycin (1%, v/v). All cells were cultured at 37 °C in 5% CO_2.

Synthesis of MSNs/HAP. Cetyltrimethylammonium bromide (CTAB, 1 g) and triethanolamine (TEA, 0.04 g) were dissolved in 20 ml deionized water under stirring. And then, pH value of mixture was adjusted to 11 by ammonia water. The mixture solution was heated to 85 °C with intensive stirring and kept in this temperature for 1.5 h. Then, the water solution of calcium nitrate [$Ca(NO_3)_2 \cdot 4H_2O$, 0.118 g], was

added dropwise under stirring for 30 min to intensive mixing. After the above steps, 1 mL of TEOS and diammonium hydrogen phosphate [$(NH_4)_2HPO_4$, 0.044 g] were added into the solution with a distinct process phenomenon from clear to opaque. The mixed solution was further reacted at 90 °C for another 24 h. In order to remove the CTAB, the obtained products were suspended in CH_3OH/HCl solution (methanol: 50 mL; 12 M hydrochloride acid: 0.25 mL) for 8 h, and collected by centrifugation and washed with EtOH/H_2O several times to remove the residual reagents (denoted as MSNs/HAP).

Synthesis of MSNs/HAP-NH_2. APTES (100 mg, 0.452 mmol) was added to 30 mL EtOH/H_2O mixed solvent (v/v: 9:1) at fully stirring for 2 h. After homodisperse of APTES, MSNs/HAP (300 mg) was added, allowing nanoparticles to be well dispersed. During the process, the pH value was maintained at ~10 by adding ammonia water. The above mixture was stirred at r.t. for another 3 h. The obtained precipitate was washed with EtOH (anhydrous) three times to remove the unreacted APTES. Then, the nanoparticles were collected through centrifugation and vacuum drying (denoted as MSNs/HAP-NH_2).

Synthesis of DOX@MSNs/HAP-NH_2. MSNs/HAP-NH_2 (100 mg) was dispersed into DOX solution (5 mg/mL, 2 mL) to kept at intensive stirring for 24 h at *r.t.*. Then the drug-loaded nanoparticles were obtained by centrifugation, and washed with ethanol (20 mL) and distilled water (20 mL) three times, respectively. The drug-loaded nanoparticles were collected through vacuum drying (denoted as DOX@MSNs/HAP-NH_2).

Synthesis of oHA-DOX@MSNs/HAP and HA-DOX@MSNs/HAP. The activation of carboxyl groups in HA (oHA: 85 mg; HA: 85 mg) was achieved through the catalysis of EDCl (30 mg) in deionized water (50 mL) for 0.5 h at r.t. N-hydroxysuccinimide (NHS, 35 mg, 0.30 mmol) was then added and kept stirring for another 12 h. The NHS-activated HA further reacted with DOX@MSNs/HAP-NH_2 (100 mg) in aqueous phase (PBS, 7.4, 50 mL) for 3 h. The products oHA-DOX@MSNs/HAP and HA-DOX@MSNs/HAP were washed with ethanol (20 mL) and distilled water (20 mL) three times to remove unreacted chemicals, respectively. The final nanoparticles were collected through centrifugation and vacuum drying.

Calculation of DOX loading amount. DOX loading amount in nanoparticles was calculated by using DOX standard UV curve. Specifically, DOX@MSNs/HAP (50 mg) was added into 25 mL acidic PBS buffer (pH = 2) with fully agitation for 24 h. The obtained solution gradually turned to be clear red, suggesting that the structure of MSNs/HAP were entirely destroyed and the loaded drugs (DOX) were released thoroughly. The DOX loading content was calculated by using the standard UV curve ($[A] = 0.0472[c_{DOX}] + 0.0301$).

Calculation of surface-grafted HA. A traditional HA detection method by using carbazole to determine the amount of surface-grafted HA. oHA-DOX@MSNs/HAP (or HA-DOX@MSNs/HAP) (30 mg) was added into 5 mL $NaBH_4$-H_2SO_4 solution (0.025 mol/L) at 0 °C, and the obtained solution was kept at sufficient oscillation to complete the reaction. Next, the solution was heated to 100 °C and kept for 20 min, and cooled down to r.t. followed by treating with 0.2 mL carbazole detection kits.

Then, the solution was kept again at 100 °C water for 15 min and cooled to r.t. to measure the absorption value at 530 nm. The HA content was calculated by using the regression equation which was from the detection kits' specification.

$$[Abs_{530}] = 0.00145674 \times C_{HA} + 0.0076982$$

Where C_{HA} represents HA concentration; Abs_{530} represents absorption intensity at 530 nm.

Thus, HA-grafting capacity was obtained through the equation:

$$HA_{grafting\ capacity} = \frac{M_{Agent}}{M_{Total}} \times 100\ \%$$

Where M_{Agent} represents the amount of HA that grafted to the nanoparticles and M_{Total} represents the amount of (o)HA-DOX@MSNs/HAP.

Characterization of HA-DOX@MSNs/HAP. DLS analysis (size & PDI value) and ζ-potential of nanoparticles were measured by an ethanol electrophoresis method using a Zetasizer Nano Zetasizer Nano ZS90. All measurements were means of three separate measurements.

Prior to TEM examination, these samples were preliminary treated with 2 % v/v phosphotungstic acid for 5 min, and then deposited on the carbon mesh with a drop liquid, and removed the excess sample with filter paper and dried at 25 °C.

The morphology of samples was observed in Tecnai F30 TEM (FEI, America) in the voltage of 300 kV. Besides, the SEM experiments were carried out in SEM (NOVA NanoSEM 450, FEI, America).

Drug Release Profile. Releasing behavior of drugs from DOX@MSNs/HAP was measured by UV−vis spectroscopy. Different acidic PBS (pH: 7.4, 6.5, 6.0 and 5.5) were prepared to stimulate the blood (~ 7.4), cancer site (including cells and its

interstitial fluid, ~ 6.5), endosomes (6.0 ~ 6.5) and lysosome (~ 5.5). DOX@MSNs/HAP nanoparticles were dispersed in the above solutions at 37 °C with gentle agitation. Drug release was monitored by UV−vis spectroscopy and repeated three times (n = 3).

Cytotoxicity experiments. 3-(4,5-dimethylthiazol-2-yl)-2,5-diphenyltetrazo lium bromide (MTT) experiments were carried out to measure the cytotoxicity. Cells (1 × 10^4 cells per well) were seeded in microplates (96-well, Corning, America). Each well contained 100 μL medium or PBS. After 24 h, the cells were washed with 100 μL/well PBS and then cultured in medium with different drug content (0.08, 0.17, 0.34, 0.675, 1.25, 2.5, 5, 10 and 20 mg DOX/L) of nanoparticles for 24 h. The control group (set as 100 % cell viability) were cultured in the medium without any treatment. Every control and test concentration were set up with six replicate wells. Then MTT (5 mg/mL, 100 μL) was added to each well and the 96-well microplates were incubated at 37 °C. After 4 h incubation, the medium was carefully removed, and 200 μL DMSO was added to lyse the purple crystals. The OD (optical density) value was measured by using a microplate reader (Thermo Fisher Scientific) at 490, 570, 630 nm. The cell viability was calculated using the following equation:

$$Cell\ viability\ \% = \frac{OD_{sample} - OD_{blank}}{OD_{control} - OD_{blank}} \times 100\ \%$$

Where OD represents the absorbance value at 490 nm, 570 nm, 630 nm.

Flow Cytometry. An Attune NxT flow cytometer (Thermo Fisher Scientific) was used in flow cytometry test. To quantify the CD44 expression in cell membrane, four cell lines (COS-7, MCF-7, HepG2, 4T1) were incubated with

oHA-DOX@MSNs/HAP or HA-DOX@MSNs/HAP. Generally, cells (2×10^5) were seeded into six-well plates one day before use. The two wells were as control to determine the original CD44 protein content of cells, the two wells were incubated with oHA-DOX@MSNs/HAP (5 mg DOX/L), and the other right two were incubated with HA-DOX@MSNs/HAP (5 mg DOX/L). A commercial CD44-PE antibody was used to measure the CD44 content (λ_{ex} = 545 nm, λ_{em} = 590 nm). After incubation for 12 h, the cells were washed twice with PBS solution before flow cytometry (Attune NxT) analysis.

Western-blot assay. The content of CD44 protein in cell membrane was tested by a series of western-blot experiments. The cells were washed with cold PBS three times followed by trituration in Triton X-100 (1%) and PMSF (1 mM) for 1 h adequately. The whole procedure described above was performed on ice strictly. With freeze-drying methods, the secreted proteins were acquired and enriched from the extracellular medium. Pellet cell debris was collected by protein lysates, and the supernatant was obtained and quantified by Lowry protein assay. Thereafter, samples were eluted with SDS buffer, separated on SDS polyacrylamide gels, and electroblotted onto PVDF membranes (Bio-Rad, USA). Membranes were blocked in 5 % nonfat milk for 2 h and incubated with primary antibodies to CD44. All antibodies were diluted 1000 folds with incubation overnight. HRP-conjugated IgG secondary antibodies (ZSGB-BIO, Beijing, China) were used to incubate with membranes for 1 h. Western Lightning Plus-Enhanced Chemiluminescence Substrate (PerkinElmer, Inc., Waltham, MA, U.S.A.) was used to visualize protein bands.

Live cell imaging experiments. For each cell line, the cells were seeded in a cover

glass-bottomed culture well with a number of 5×10^4, and then incubated at 37 °C for 24 h. HA-DOX@MSNs/HAP or oHA-DOX@MSNs/HAP (5.0 mg DOX/L) were added, and cells were further incubated for 120 min or 240 min. Cell imaging experiments were carried out by using OLYMPUS FV-1000 inverted fluorescence microscope with a 100 × objective lens. Cells were excited at two laser wavelength (405 nm, 488 nm), and the emission was collected at 430-470 nm (Hoechst 33342: blue channel) and 590-620 nm (DOX: red channel), respectively

Hemolysis experiments. We collected about 1 mL of fresh blood from female Bal b/c nude mice through centrifugation at a speed of 2, 000 × g for 3 min. Red blood cells (RBCs) were isolated and then washed with PBS for several times until no red color can be observed from the supernatant. Eight groups (negative and positive control groups, and samples incubated with oHA-DOX@MSNs/HAP at six different concentrations) were prepared: RBC (0.10 mL) mixed with 0.90 mL PBS or 0.90 mL distilled water were used as the negative and positive control groups, respectively; a mixture of 0.10 mL of the RBC and 0.90 mL PBS containing oHA-DOX@MSNs/HAP nanoparticles (final concentrations: 1.3, 2.5, 5.0, 10, 20, 40 μg/mL). After gently shaking, these samples were intensive mixed, which were left at r. t. for 2 h, followed by centrifugation at 2,000 × g for 3 min to precipitate the red cells. To determine the absorbance of hemoglobin, we took 100 μL of every supernatant to a 96-well plate, and detected the absorbance changes at 540 nm. The hemolysis was evaluated via the following equation:

$$Hemolysis\ \% = \frac{A_{sample} - A_{negative\ control}}{A_{positive\ control} - A_{negative\ control}} \times 100\ \%$$

Where A represents the absorbance value at 540 nm.

Cell apoptosis experiments. HepG2, 4T1 and COS-7 cells (approximately 2 × 10^5 cells) were seeded in a 6-well plate. After incubation for 24 h, the medium was removed and new fresh DMEM medium containing special concentrations of free DOX, DOX@MSNs/HAP, oHA-DOX@MSNs/HAP and HA-DOX@MSNs/HAP were added to each well of the plate, which was further incubated overnight. Afterwards, cells were collected and washed with cold PBS (three times). The collected cells were suspended in binding buffer (400 μL), stained with apoptosis staining kit (5 μL of annexin V–FITC and 10 μL of PI). Cell apoptosis imaging experiments were carried out by using OLYMPUS FV-1000 inverted fluorescence microscope with a 100× objective lens. Three laser wavelength (458 nm, 488 nm, 559 nm) were selected, and the emission was collected at 510-530 nm (AV-FITC: blue channel), 590-610 nm (DOX: red channel) and 600-630 nm (PI: purple channel). The apoptosis effect in cells was finally determined by analyzing 1×10^4 cells with FCM (flow cytometer, Thermo Fisher Scientific).

Anticancer efficiency in vivo. The tumor-bearing BALB/c mice were originally purchased from SPF experimental Animal Center of Dalian Medical University. This study was conducted in accordance with the Guide for the Care and Use of Laboratory Animals published by the US National Institutes of Health (8th edition, 2011). The animal protocol was approved by the local research ethics review board of the Animal Ethics Committee of Dalian University of Technology (Certificate number//Ethics approval no. is 2018-043). Xenograft tumors were established in female athymic nude mice (6-7 weeks old, 14-18 g) by subcutaneous injecting of 1×10^6 4T1 cells to left

flanks. After about two weeks, when the tumor volume reached 86 mm^2, mice were divided into four groups randomly (five mice per group) to conduct with different treatments. All mice received a tail vein injection with administration of either 200 μL PBS (control group), 5 mg/kg free DOX, 5 mg DOX/kg of DOX@MSNs/HAP, 5 mg DOX/kg of HA-DOX@MSNs/HAP or 5 mg DOX/kg of oHA-DOX@MSNs/HAP nanoparticles，respectively. The tumor volume was measured every 3 days and calculated using the formula $V = (a \times b^2)/2$, where a and b are the longest and shortest length of tumors, respectively. Tumor growth in the different treatment groups was monitored. Besides, the body weights were also recorded during the treatment. After treatment, all mice were scarified for further biosafety evaluation. H&E staining was carried out by Beijing Lawke Health Laboratory Center for Clinical Laboratory Development**.** Images were taken using a light microscopy EVOS XL Core (Thermo Fisher, USA).

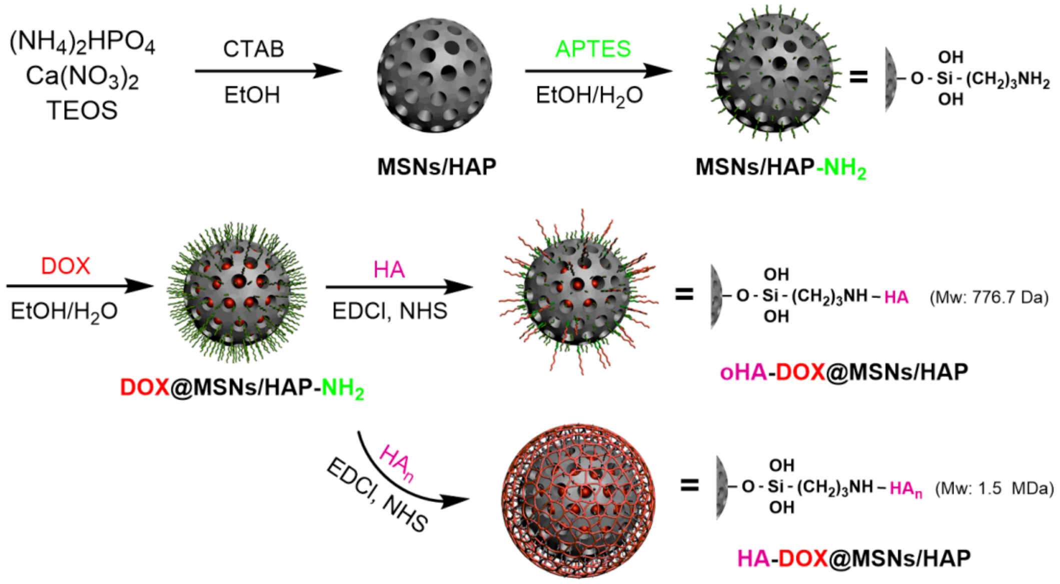

Scheme S1. Preparation scheme of oHA-DOX@MSNs/HAP and HA-DOX@MSNs/HAP.

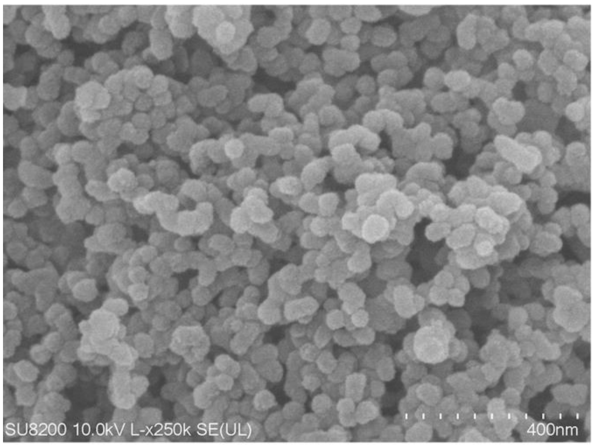

Figure S1. SEM image of MSNs/HAP.

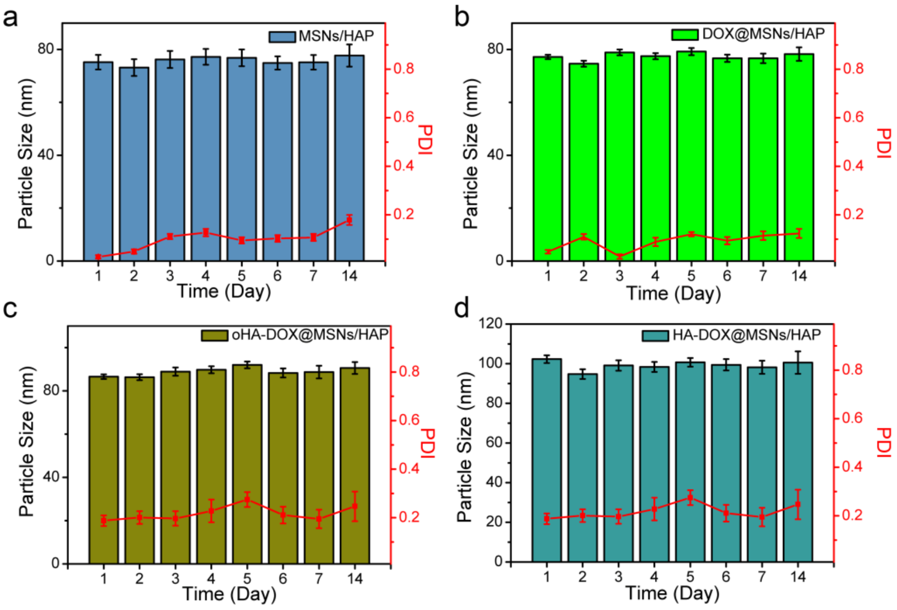

Figure S2. Nanoparticle size and PDI changes of MSNs/HAP, DOX@MSNs/HAP, oHA-DOX@MSNs/HAP and HA-DOX@MSNs/HAP within 2 weeks (n = 3).

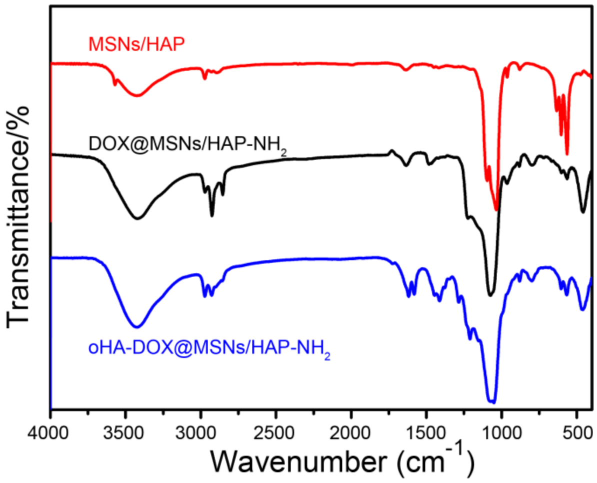

Figure S3. FT-IR spectra of MSN/HAP, DOX@MSNs/HAP-NH_2, and oHA-DOX@MSNs/HAP.

Table S1. XPS analysis of MSNs/HAP

Element	App conc.	Intensity Corrn.	Weight %	Weight %	Atomic %
O K	7.91	0.0855	68.28	0.55	25.72
Si K	10.96	0.2656	30.45	0.23	10.04
Ca K	0.73	0.7908	0.69	0.02	0.13
P K	0.66	0.8741	0.58	0.02	0.11
Total			100.00		

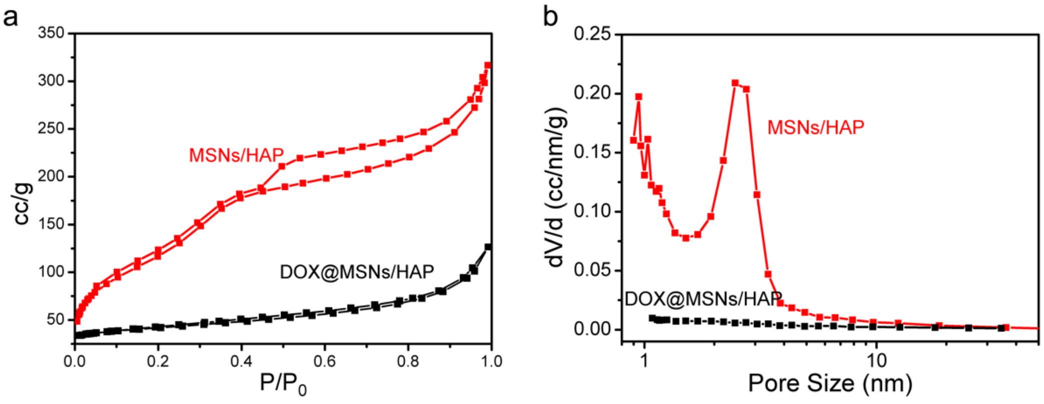

Figure S4. (a) N_2 adsorption-desorption isotherms of DOX@MSNs/HAP and MSNs/HAP. (b) the pore distribution of DOX@MSNs/HAP and MSNs/HAP.

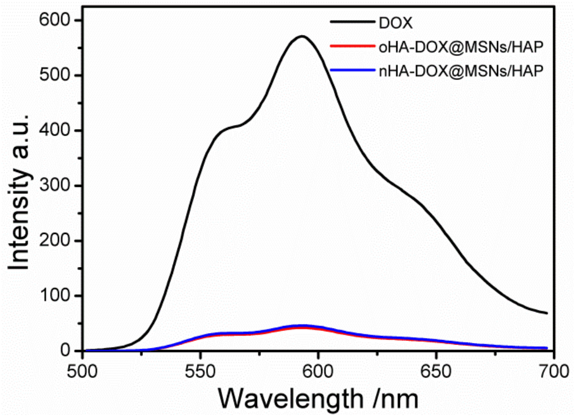

Figure S5. Fluorescent spectra of oHA-DOX@MSNs/HAP, HA-DOX@MSNs/HAP and DOX.

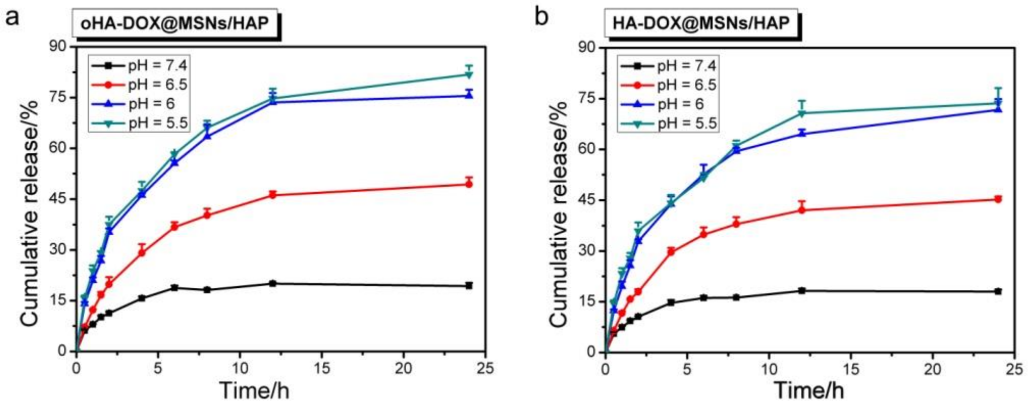

Figure S6. Release profiles of DOX from oHA-DOX@MSNs/HAP (a) and HA-DOX@MSNs/HAP (b) in PBS buffer with different pH values.

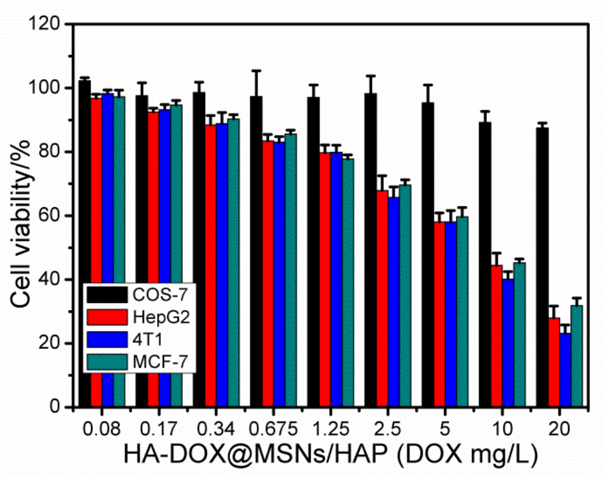

Figure S7. Viability of normal cells (COS-7) and cancer cells (HepG2, MCF-7 and 4T1) treated with HA-DOX@MSNs/HAP at various concentrations (n = 4).

Table S2. IC_{50} value of oHA-DOX@MSN/HAP and HA-DOX@MSNs/HAP in cancer and normal cells (unit: mg DOX/L)

IC_{50} (DOX mg/L)	MCF-7	4T1	HepG2	COS-7
oHA-DOX@MSNs/HAP	4.44	3.84	4.04	N/A
HA-DOX@MSNs/HAP	7.55	7.06	7.46	N/A

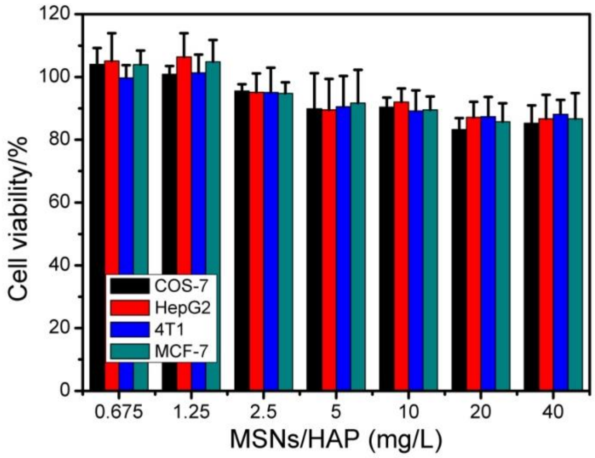

Figure S8. Viability of normal cells (COS-7) and cancer cells (HepG2, MCF-7 and 4T1) treated with MSNs/HAP at various concentrations (n = 4).

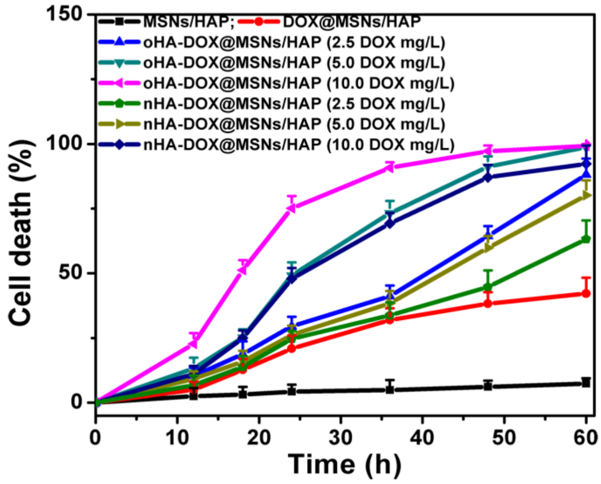

Figure S9. Cell-death percentages of 4T1 cells after different treatments (n = 3).

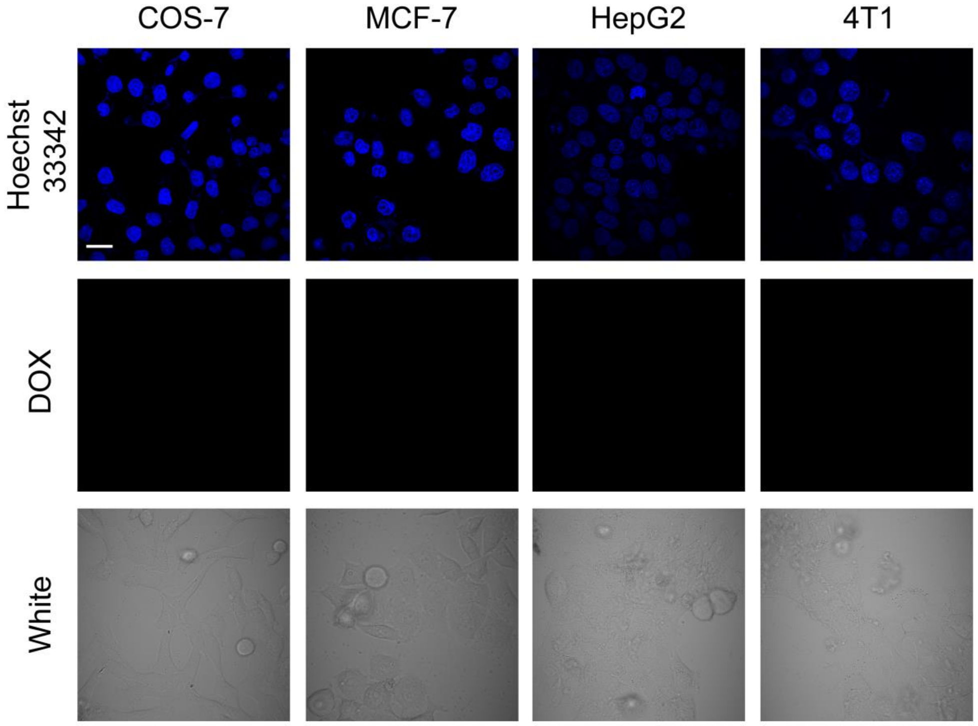

Figure S10. CLSM images of cells after incubation Hoechst 33342 (λ_{ex} = 405 nm, λ_{em} = 430-470 nm) and DOX channel (λ_{ex} = 488 nm, λ_{em} = 590-620 nm)as the control group. Scale bar: 20 μm.

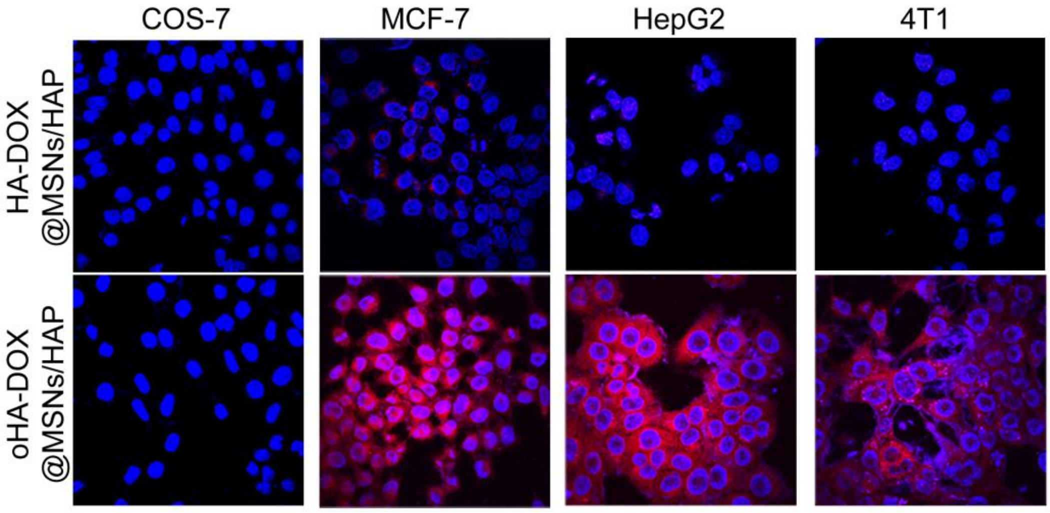

Figure S11. CLSM images of cells treated with different nanoparticles for 120 min. Blue: Hoechst 33342 (λ_{ex} = 405 nm, λ_{em} = 430-470 nm); Red: DOX (λ_{ex} = 488 nm, λ_{em} = 590-620 nm).

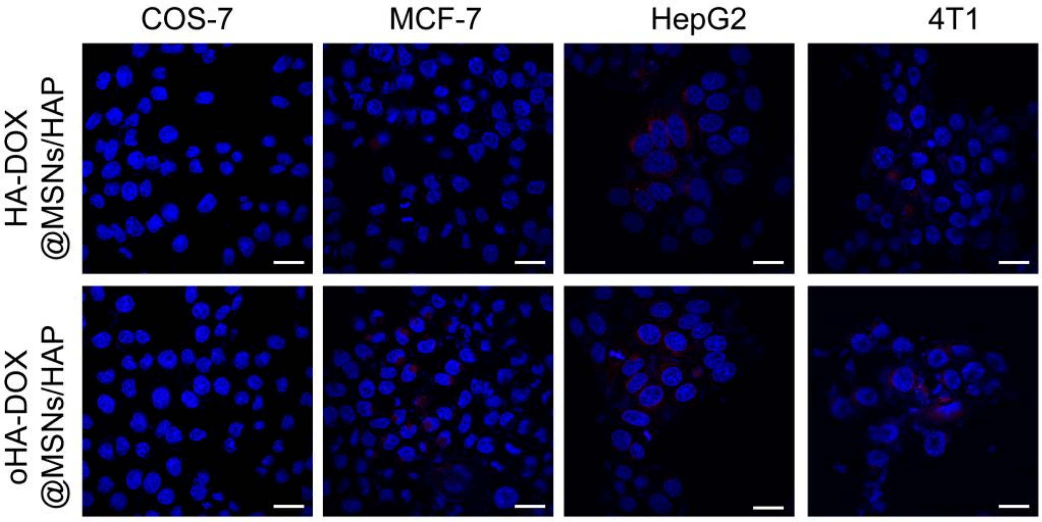

Figure S12. CLSM images of cells treated with different nanoparticles for 4 h (pretreated by CD44 monoclonal antibody). Blue: Hoechst 33342 (λ_{ex} = 405 nm, λ_{em} = 430-470 nm); Red: DOX (λ_{ex} = 488 nm, λ_{em} = 590-620 nm).

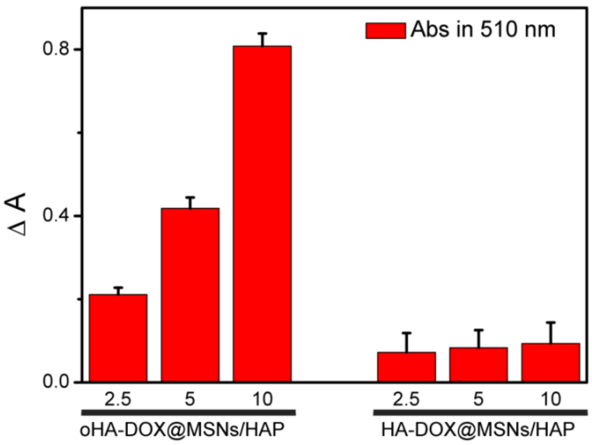

Figure S13. Cellular uptake of oHA-DOX@MSNs/HAP and HA-DOX@MSNs/HAP after 24 h incubation (2.5, 5, 10 mg/L). Alcian blue was used to measure the HA amount in intracellular (detected in cell lysis buffer).

Table S3. FCM data analysis of cancer and normal cells after different treatment.

	MCF-7		4T1		HepG2		COS-7	
Control	9873	Positive	24398	Positive	159177	Positive	7927	
HA-DOX@MSNs/HAP	1552	Positive	7645	Positive	4157	Positive	8209	No Changes
oHA-DOX@MSNs/HAP	46.2	Negtive	45.8	Negtive	61.1	Negtive	2827	

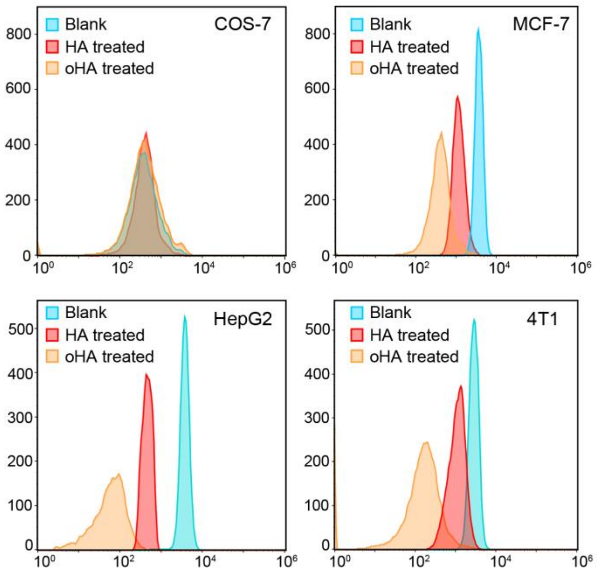

Figure S14. CD44 content in cancer and normal cells after different treatments determined by using CD44-PE monoclonal antibody. Blue: control; Red: incubation with HA for 6 h; Yellow: incubation with oHA for 6 h.

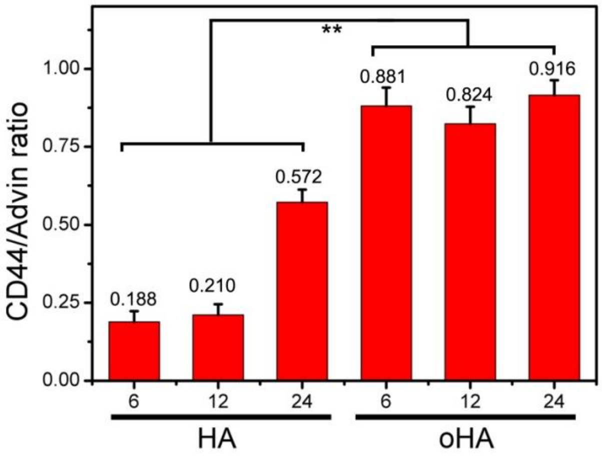

Figure S15. Data analysis of "Pull-Down" experiment, demonstrating the interactions between oHA/HA with CD44 by using specific biotin and avidin interaction. Time: 6 h, 12 h, 24 h. (**: $p < 0.005$, Tukey's multiple comparison test)

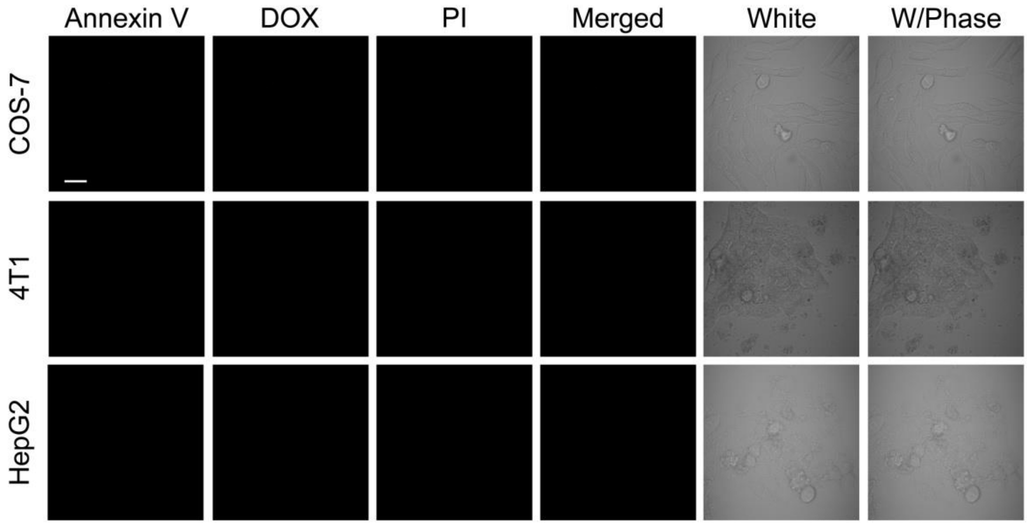

Figure S16. CLSM images of COS-7, 4T1 and HepG2 cells after incubation with the AV-PI (Annexin V-FITC and Propidium Iodide) staining as the control group. Scale bar: 20 μm.

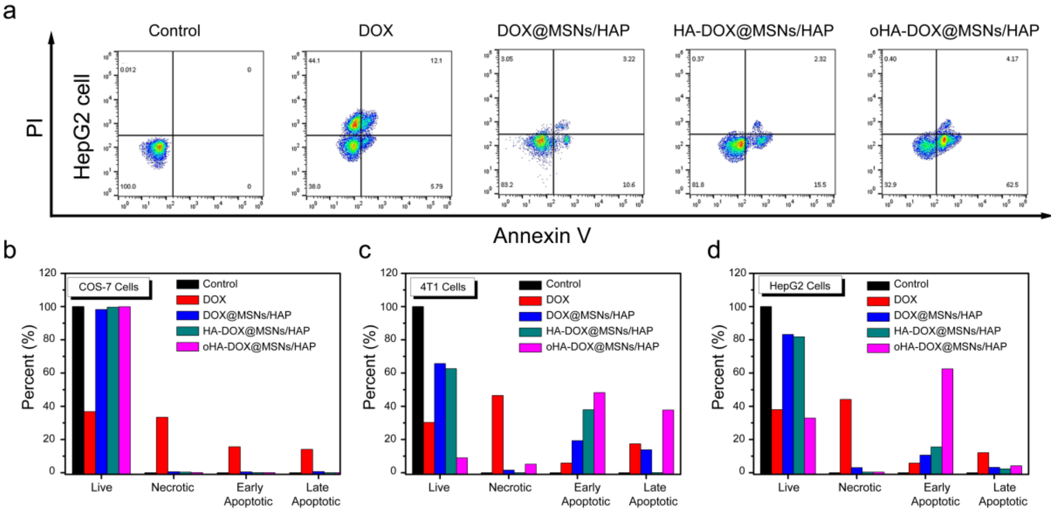

Figure S17. (a) Flow cytometric examination of drug-induced apoptosis HepG2 cells after incubation with DOX, DOX@MSNs/HAP, HA-DOX@MSNs/HAP and oHA-DOX@MSNs/HAP for 24 h. (b) Data analysis of FCM results of COS-7 (Fig. 4b), 4T1 (Fig. 4b) and HepG2 cells (Fig. S10a).

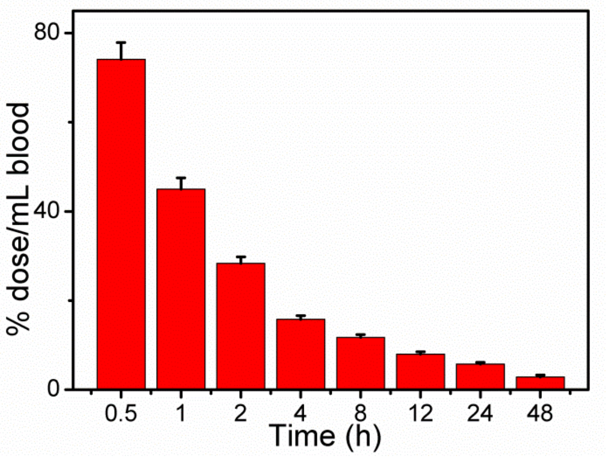

Figure S18. The change of Si concentration in blood after treatment with oHA-DOX@MSNs/HAP (n = 3)

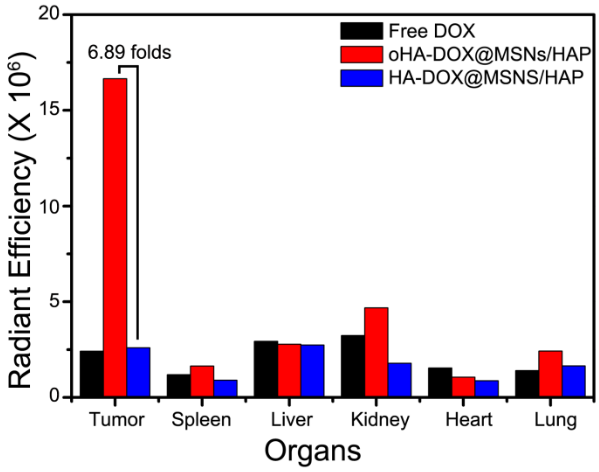

Figure S19. Fluorescence intensity of DOX in the organs of 4T1 tumor-bearing mouse collected at 24 h after injection of free DOX, oHA-DOX@MSNs/HAP and HA-DOX@MSNs/HAP nanoparticles.

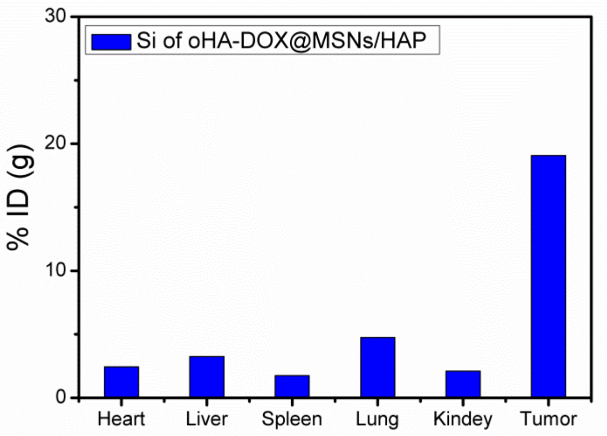

Figure S20. ICP-MS analysis of Si amount in the organs of 4T1 tumor-bearing mouse collected at 24 h after injection of oHA-DOX@MSNs/HAP. The data analysis of Si amount was in accordance with DOX intensity measurement (**Figure S14**), indicating that the major location and drug release were accumulated in tumor sites.

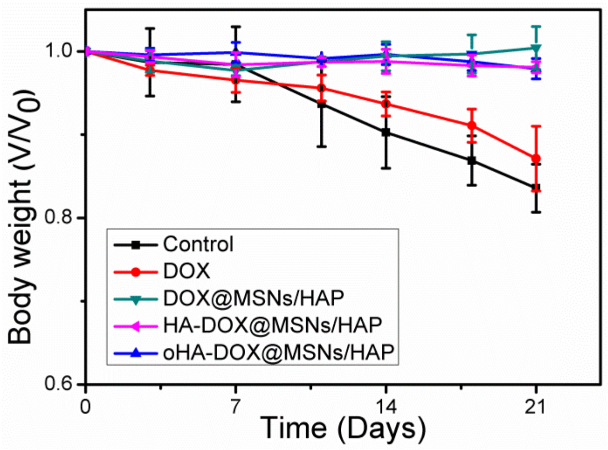

Figure S21. Body weight of tumor-bearing mice from different groups (n = 5). The body weight of the mice treated with DOX@MSNs/HAP, HA-DOX@MSNs/HAP, oHA-DOX@MSNs/HAP were stable during treatment, indicating that the injection of nanoparticles had minimal side effects on the mice.

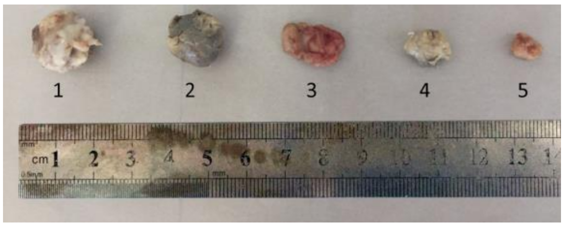

Figure S22. Photographic images of excised tumors after treatment. (1. Control; 2. DOX; 3. DOX@MSNs/HAP; 4. HA-DOX@MSNs/HAP; 5. oHA-DOX@MSNs/ HAP)

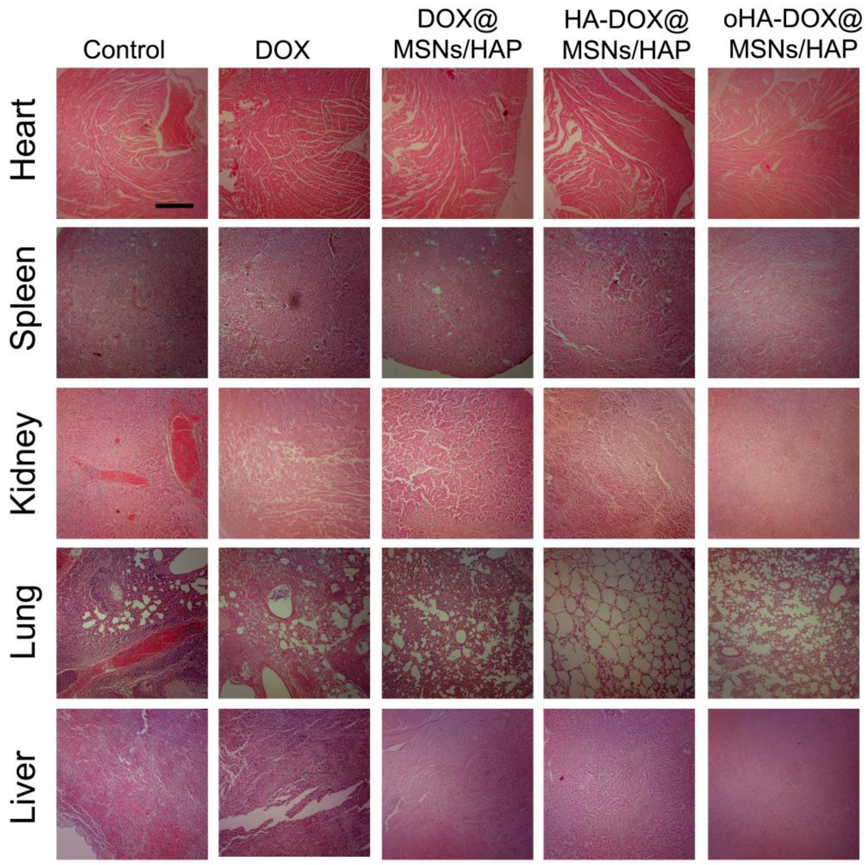

Figure S23. H&E staining of organ tissues from different groups.

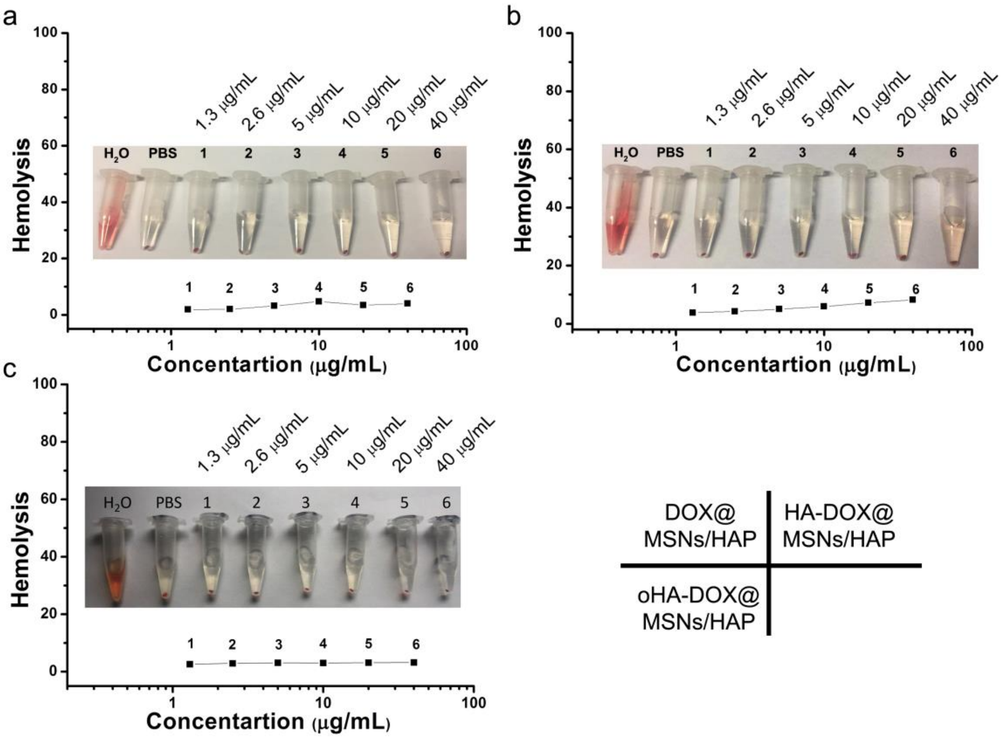

Figure S24. Blood hemolysis after incubation with DOX@MSNs/HAP (a), HA-DOX@MSNs/HAP (b) and oHA-DOX@MSNs/HAP (c) at different concentrations (sample 1 to 6) for 2 h. No hemolysis was observed even when the concentration was 40 μg/mL, demonstrating that the nanoparticles are safe to the blood.

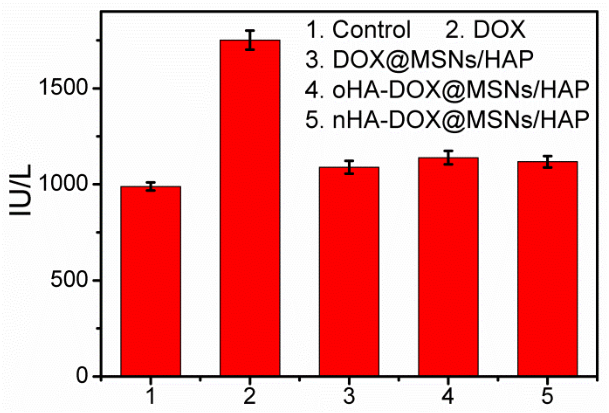

Figure S25. The changes of serum CK levels of serum samples on 4T1 bearing mice after different treatment (n = 3). The CK level of the mice treated with DOX@MSNs/HAP, HA-DOX@MSNs/HAP, oHA-DOX@MSNs/HAP were stable during treatment, indicating that the injection of nanoparticles had minimal damage to hearts, livers and spleens. All above tests are kept at the concentration of 5 mg DOX/L.
